# Cancer of Unknown Primary: A Review on Clinical Guidelines in the Development and Targeted Management of Patients with the Unknown Primary Site

**DOI:** 10.7759/cureus.5552

**Published:** 2019-09-02

**Authors:** Aisha Qaseem, Norina Usman, Joseph S Jayaraj, Rajesh Naidu Janapala, Tooba Kashif

**Affiliations:** 1 Internal Medicine, California Institute of Behavioral Neurosciences and Psychology, Fairfield, USA; 2 Internal Medicine, Veterans Affairs Palo Alto Health Care System - Stanford University School of Medicine, Palo Alto, USA; 3 Internal Medicine, Icahn School of Medicine at Mount Sinai/Queens Hospital Center, New York, USA

**Keywords:** cancer of unknown primary site, cancer of unknown primary, primary cancer of unknown origin, metastatic cancer of unknown primary site

## Abstract

Cancer of unknown primary (CUP) is a malignant widespread metastatic disease without an identifiable primary site after extensive clinical investigation. Recently, a decline is observed in the diagnosis of CUP, mainly due to improvement in detection of the primary tumors, thus decreasing the unknown primaries. Worldwide, CUP is the sixth to eighth most common malignancy, accounting for 2.3% to 5% of a new cancer diagnosis. CUP is third to fourth most common cause of death due to cancer-related mortality. The prognosis of CUP is depressing with the median survival of three to six months in the previous studies, but according to recent studies, median survival is less than one year. High risk for developing CUP is seen in heavy smokers (26 or more cigarettes/day) and individuals with the lowest quartiles of waist circumference. A weak association is observed with the use of alcohol consumption and low level of education. Human papillomavirus DNA plays a role in those with squamous cell carcinoma of unknown primaries in head and neck regions. In the diagnosis of CUP, comprehensive medical history, complete physical examination (including genitourinary, rectal exam, and breast examination in women) and necessary laboratory tests are crucial.

Whole-body positron emission tomography-computed tomography (PET/CT) is the investigation of choice to assess the entire body for CUP. Multiparametric 3T-MRI (MP-MRI) is used to examine the local soft tissue status, helps in the staging of the tumor, and to determine the extent of involvement of tissue for medical as well as prognostic purposes. Immunohistochemistry outlines the specific markers, including caudal-related homeobox protein (CDX2), homeobox protein Nkx-3.1 (NKX3-1), paired box gene 8 (PAX8), special AT-rich sequence-binding protein 2 (SATB2), thyroid transcription factor 1 (TTF-1), and splicing factor 1 (SF1) with the focus on the effectiveness of lineage-restricted transcription factors. Patients response to treatment can be evaluated by the gene expression profiling (GEP) test that also predicts tissue of origin (TOO). Tumor identified through gene profiling is sensitive to platinum/taxane therapy, others that are not TOO tumors are resistant to platinum/taxane. The new therapeutic method based on molecular profiling is associated with higher treatment response. In comprehensive genomic profiling, it is observed that there is at least one clinically appropriate genomic alteration in CUP that can influence the targeted therapy. The targeted therapeutic approach will not only improve the disease outcome but will also be cost-effective and save time from finding the primary site.

## Introduction and background

Cancer of unknown primary (CUP) is a malignant widespread metastatic disease without an identifiable primary site after extensive clinical investigation [1]. Based on the biological features, clinical presentation, and atypical pattern of metastasis, the CUP can be considered as a separate disease from the other known primary tumors [1-2]. Hypotheses on the pathogenesis of CUP are:

1) Stem cell producing cancer without premalignant lesion or primary cancer.

2) A very early primary cancer causes a rapid progression of metastasis. 

3) Recently a possible explanation was proposed on the role of chromosomal instability contributing to aggressive disease presentation and chemoresistance [2].

In the past 50 years, the incidence of CUP has increased probably due to higher diagnostic sensitivity, thus finding more metastatic disease. Later the rate of CUP is decreased by improvement in detecting the primary tumor site, thus reducing the unknown primaries [3]. Worldwide, CUP is the sixth to eighth most common cancer, accounting for 2.3% to 5% of new malignancy diagnosed. CUP is third to fourth most common cause of death due to cancer-related disease ranking CUP among the top 10 cancers in both the genders in terms of incidence and mortality [3-5].

Here, we are trying to know the histopathology and the targeted treatment in depth that can be utilized as a guideline in the future for the diagnosis and management of CUP. More precise treatment can only be started after the specific type of cancer is diagnosed, whereas in CUP drug treatment is less effective because of the lack of specific therapies. In addition to this, the delay in diagnostic evaluation further worsens the patient condition that the therapeutic procedures cannot be implemented [5]. Studying CUP in detail will not only boost the knowledge of the researchers and physicians but also help the patients in the future.

Developing data from the studies showed an increased rate of emergency and hospital admission, psychological distress as well as decrease rate of consultations by the specialist. In patients with CUP, primary care physicians have a vital role in early diagnosis and integrated care [6].

As discussed earlier, not much is known about CUP despite having high mortality and poor prognosis. In this article, we explore the age-specific incidence, possible histopathology in the development of CUP and known risk factors. The focus of the report will be towards the diagnosis and treatment guidelines published in the recent literature up to the year 2019.

## Review

CUP represents as the most heterogeneous groups of cancers. It has the most aggressive behavior and is highly resistant to therapy. A better understanding of treatment and prognostic factors is possible by studying patients in homogenous subgroups with CUP [7]. 

Incidence, prevalence, and prognosis

Based on data from the studies, the prognosis of CUP is depressing with the median survival at three to six months in earlier studies, but according to recent studies, median survival is less than one year [8-9]. Site of metastasis and histology are two essential factors in the prognosis of CUP [10]. The incidence of CUP is highest in patients of age between 60-75 years [2]. In another study, the highest incidence ratio was at age 85-89 years, followed by a significant decrease by age ninety-plus (7-fold in men and 3-fold in women) [10].

Risk factors

To evaluate the possible risk factors for CUP, proportional hazards models were conducted to explore the associations of risks with the development of the disease. The significant risk for developing CUP was seen in current heavy smokers (26 or more cigarettes/day) compared to those who never smoked. In subjects having the highest vs. lowest quartiles of waist circumference showed a 30% increase risk of CUP [9]. A weak association was seen for alcohol consumption and low level of education [11-12]. In a study of 60 patients on head and neck CUP (squamous cell carcinoma on histology), human papillomavirus (HPV) DNA was found positive in 13 patients making 22% of the study [13]. In another study of 59 patients with a median follow up time of 5.3 years, the role of HPV and incidence of tumor suppressor protein (p16) overexpression in the development of cutaneous head and neck squamous cell carcinoma (SCC) was evaluated. HPV RNA was performed to detect 18 high-risk HPV subtypes. P16 doesn't show any difference in clinical pathology hence high-risk HPV subtypes have no association with p16 positivity and no role in the CUP [14]

Classification of CUP

The Guidelines issued by the United Kingdom National Institute of Clinical Excellence (NICE) for the management of CUP also established the classification of definitions that replicate distinct phases of investigations (Table 1) [1].

**Table 1:** Classification of definition by the National Institute of Clinical Excellence (NICE) CUP- cancer of unknown primary.

Term	Definition
Malignancy of undefined primary origin (MUO)	Limited tests showed Metastatic malignancy without any clear primary site before the comprehensive investigations are performed.
Provisional CUP (pCUP)	Selected initial tests based on cytology and histology showed metastatic epithelial or neuroendocrine malignancy without any primary site of origin, before specialist evaluation and likely after additional specialized investigations.
Confirmed CUP	Final histology showed metastatic epithelial or neuroendocrine malignancy without any primary site of origin even after the initial tests, specialist evaluation, and likely additional specialized investigations.

Diagnostic workup

The United State National Cancer Institute (NCI), the National Comprehensive Cancer Network (NCCN), and the European Society of Medical Oncology (ESMO) have published international clinical guidelines on the diagnostic investigations to perform in suspected patients with CUP in order to rule out any cancer presenting with metastasis [2]. In 2010, the National Institute of Clinical Excellence (NICE) endorsed to form a devoted multidisciplinary team of oncologist, palliative physicians, and nurse specialists for the CUP patients [1,15]. The purpose was the early diagnosis and management of the symptoms, later there was no change observed towards the improvement in the performance status or survival time in patients with CUP [15]. 

The primary clinical workup includes complete history with particular attention to previous diseases and procedures. Comprehensive physical examination, including genitourinary examination, rectal examination, and breast examination in women is essential. Necessary laboratory tests and computed tomography (CT) scan of the abdomen, chest, and pelvis are the initial investigations [2]. Positron emission tomography-computed tomography (PET/CT) and multiparametric 3T-MRI (MP-MRI) have equal accuracy in the diagnosis of CUP with metastasis of neck lymph nodes. Whole-body PET/CT is the investigation of choice to assess the whole-body status in a single examination. MP-MRI is used to examine the local soft tissue involvement after the positive PET/CT results. MP-MRI helps in tumor staging and to determine the extent of the tissue involvement for medical as well as prognostic purposes [16]. Immunohistochemistry outlines the specific markers, including caudal-related homeobox protein (CDX2), homeobox protein Nkx-3.1 (NKX3-1), paired box gene 8 (PAX8), special AT-rich sequence-binding protein 2 (SATB2), thyroid transcription factor 1 (TTF-1), and splicing factor 1 (SF1) with the focus on the effectiveness of lineage-restricted transcription factors [17]. 

Pathophysiology

Cancers are caused by oncogenesis or tumor suppressor genes mutation [2]. Pathological changes that play a role in the development of CUP includes angiogenesis activation (50%-89%), oncogene over-expression (10%-30%), significant hypoxia-related proteins and epithelial-mesenchymal transition markers (16%-25%), and the activation of intracellular signals such as protein kinase B (AKT) or mitogen-activated protein kinase (MAPK) (20%-35%) (Table 2) [2]. The possible explanation for CUP's aggressive behavior, drug resistance, and poor prognosis is chromosomal instability [18].

**Table 2:** Pathology in the development of the cancer of unknown primary (CUP) Akt - protein kinase B (PKB), MAPK- mitogen-activated protein kinase.

Pathogenesis	
Angiogenesis activation	50–89%
Oncogene over-expression	10–30%
Hypoxia-related proteins and epithelial-mesenchymal transition markers	16–25%
Activation of intracellular signals such as Akt or MAPK	20-35%

Histology

**Figure 1:**
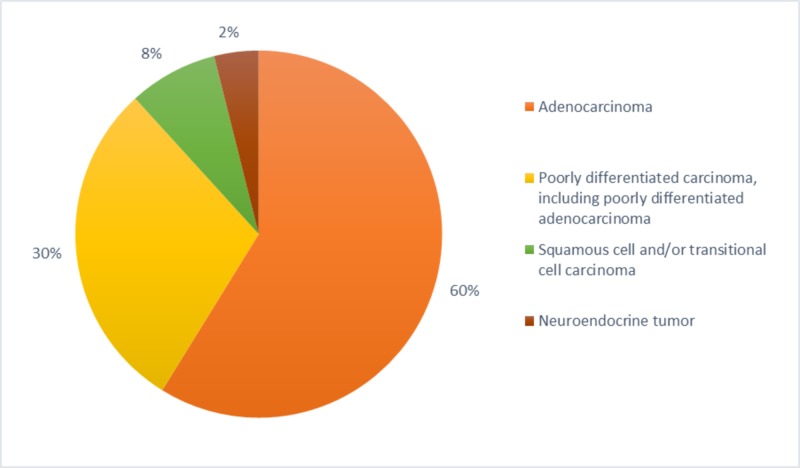
Different types of histopathological prevalence in cancer of unknown primary (CUP)

Carcinoma/neuroendocrine tumor includes adenocarcinoma, poorly differentiated carcinoma (including poorly differentiated adenocarcinoma), squamous cell and/or transitional cell carcinoma, neuroendocrine tumor, and undifferentiated carcinoma. Other less common CUP's are lymphoma, extragonadal germ cell tumor, melanoma and sarcoma [2]. Neuroendocrine carcinoma contributes to 2% - 4% and squamous cell carcinoma to 5% - 8%, these two histological subgroups show significantly better prognosis than adenocarcinoma (60%) or undifferentiated carcinomas (30%) (Tables 3-4) (Figure 1) [8,19].

**Table 3:** Initial classifications of cancer of unknown primary (CUP) include carcinoma/neuroendocrine tumor

Histopathological Types of CUP	Prevalence
Adenocarcinoma	60%
Undifferentiated carcinoma, poorly differentiated carcinoma, including poorly differentiated adenocarcinoma	30%
Squamous cell and/or transitional cell carcinoma	5%-8%
Neuroendocrine tumor	2%-4%

**Table 4:** Other histopathological types of cancer of unknown primary (CUP)

Others
Lymphoma
Extragonadal germ cell tumor
Melanoma
Sarcoma

Metastasis

Multiple metastases at presentation are common involving liver, abdomen, brain, thorax, bones, and lungs [10]. Peak incidence in abdomen and liver metastasis is seen in age 80-89-year-old individual. Thoracic metastasis peak at age 90-95, CUP with bone and brain metastasis peak in slightly younger age groups. Bone metastasis is the most common initial presentation accounts about 7.8%-21.7% of patients with CUP with the median survival time of 11 months [20]. In these patients, delay in the diagnosis increases their risk of skeletal-related events (SRE), such as fractures and spinal cord compressions [20]. After the age of 95+ incidence of all metastasis declines sharply [10]

Treatment

Treatment of neuroendocrine tumors is specific to the protocol, whereas squamous cell CUPS are treated as head and neck tumors of primary origin. Adenocarcinoma and undifferentiated CUPs are managed by the combination of two drugs, a platinum-based and a more specific protocol for special cases - which account for very few cases. [8]

**Figure 2:**
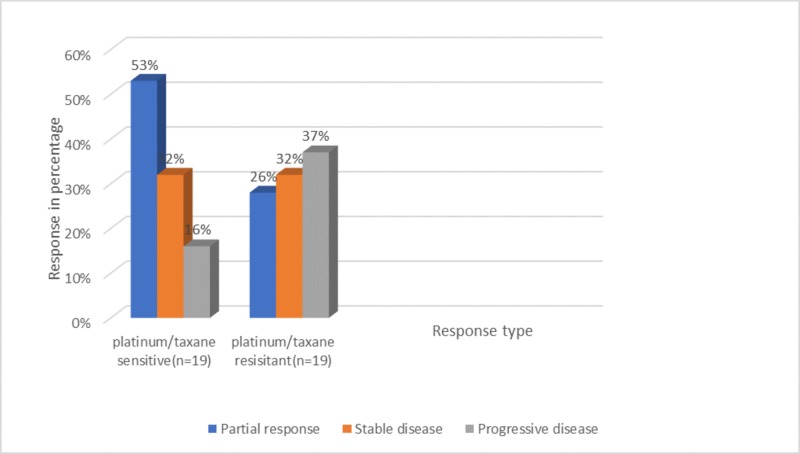
Tumor identified through gene profiling showing drug sensitivity and resistant response

Empirical treatment of CUP with platinum/taxane or platinum/gemcitabine have response rates (RR) of 15%-20% and nine months of survival [21]. The Standard treatment of CUP is carboplatin (C) and paclitaxel (P). In a phase 2 trial Everolimus, mammalian target of rapamycin (mTOR) inhibitor is added with carboplatin and paclitaxel standard treatment of CUP. Gene expression profiling (GEP) test that predicts tissue of origin (TOO) was also evaluated in the study to find the patient response [21]. Tumor identified through gene profiling is sensitive to platinum/taxane therapy, others that are not TOO tumors are resistant to platinum/taxane. It was observed that the vulnerable group showed higher tumor response rate (53% versus 26%), more prolonged progression-free survival (6.4 versus 3.5 months) and overall survival (17.8 versus 8.3 months) (Figure 2) [21].

**Figure 3:**
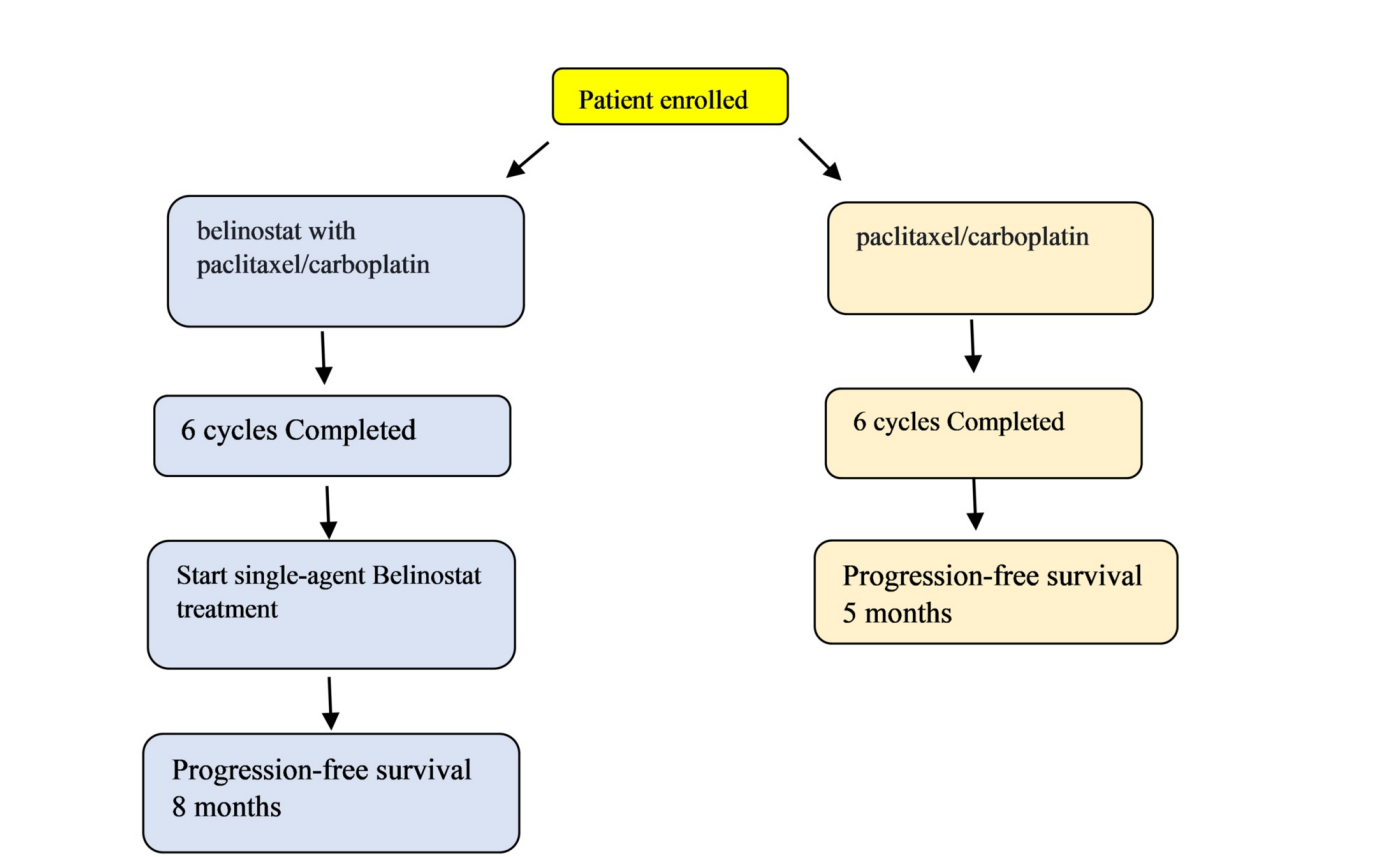
Flow chart showing progression-free survival on treatment with and without belinostat

In another randomized phase 2 trial, patients previously untreated were given belinostat with paclitaxel/ carboplatin every 21 days for six cycles. Later continued belinostat alone to the end of progression-free survival (PFS) based on reevaluation every two weeks. The results showed an improvement of PFS from five months on paclitaxel/carboplatin to eight months (Figure 3) [22].

A new therapeutic method based on molecular profiling associated with treatment benefit in 96% of the cases is proposed. Hence, expressing the wide-range of biomarker profiling using gene sequencing, immunohistochemistry, and in situ hybridization method identifies biomarkers [23]. In comprehensive genomic profiling, it is observed that there is at least one clinically appropriate genomic alteration in CUP that can influence the targeted therapy [24]. This help in choosing the specific drug and expected drug response, subsequently improving the survival [23-24]. The diagnosis and treatment of CUP are challenging due to its late presentation, difficulty in diagnosis, and therapeutic resistance as compared to other malignancies [25].

Markers for prognosis

As the prognosis of CUP is poor, clinical and laboratory tests are considered to predict the mortality. In one study it is recommended that the lactate dehydrogenase (LDH), performance studies, albumin, and a number of metastatic sites are a useful tool to assess the prognosis. Recently, clinicopathologic subgroup and leukocytosis are also considered as independent indicators of negative prognosis [25]. The modified Glasgow Prognostic Score (mGPS) is a combination of C-reactive protein (CRP) and serum albumin used in confirming the prediction of multiple cancers, including pulmonary, gastrointestinal, and renal tumors. It is observed that mGPS and neutrophil-lymphocyte ratio (NLR) correlate with the survival and prognostic accuracy in CUP [26].

## Conclusions

The diagnosis and treatment of CUP are challenging due to its late presentation, difficulty in diagnosis, and therapeutic resistance as compared to other malignancies. Precisely, the prognosis of CUP is poor and treatment choices offered are limited and non-selective. Extensive investigations have developed to identify the origin of the tissue and possible primary site by using gene expression arrays and immunohistochemistry from the biopsy site. Lately, pathway-specific therapies independent of the lineage of the membrane have been offered by identification of shared, alteration in tumor pathway from various primary sites. The targeted therapeutic approach will not only improve the disease outcome but will be cost-effective and save time from the search of the primary site. Additional studies on CUP are necessary in the future to understand the disease more clearly and to answer the following questions regarding CUP. Why CUP patients do not present early in the course of the disease?, what are the risk factors in the development of CUP?, why CUP patients remain asymptomatic for a longer duration? and how effective can targeted molecular therapy be in decreasing the mortality and improving the survival period?
